# Ultrahigh-Field MR (7 T) Imaging of Brain Lesions in Neuromyelitis Optica

**DOI:** 10.1155/2013/398259

**Published:** 2013-01-27

**Authors:** Ilya Kister, Joseph Herbert, Yongxia Zhou, Yulin Ge

**Affiliations:** ^1^Multiple Sclerosis Care Center, Department of Neurology, NYU School of Medicine, New York, NY 10016, USA; ^2^Department of Radiology, NYU School of Medicine, New York, NY, USA

## Abstract

*Background*. Brain lesions are common in neuromyelitis optica spectrum disorder (NMOsd) and may resemble lesions of multiple sclerosis (MS). *Objectives*. To describe the imaging characteristics of supratentorial lesions in NMOsd on ultrahigh-field (7 T) MRI with special attention to vessel-lesion relationship. *Methods*. Ten NMOsd patients, all women and all seropositive for NMO IgG, with mean age of 51.3 ± 15.4 years and disease duration of 9.2 ± 6.4 years, were scanned at a 7 T whole-body human MR system with high-resolution 2D gradient echo sequence optimized to best visualize lesions and venous structures, T2- and T1-weighted imaging. *Results*. In 10 patients with NMOsd, a total of 92 lesions were observed (mean: 9.2 ± 8.8; range: 2–30), but only 8 lesions (9%) were traversed by a central venule. All lesions were <5 mm in diameter, and 83% were located in subcortical white matter. There were no lesions in the cortex or basal ganglia. Two patients exhibited diffuse periependymal abnormalities on FLAIR. *Conclusions*. Small, subcortical lesions without a central venule are the most consistent finding of NMOsd on 7 T MRI of the brain. Ultrahigh-field imaging may be useful for differentiating between NMOsd and MS.

## 1. Introduction

Neuromyelitis optica (NMO) has been traditionally considered a predominantly opticospinal disorder, but recent studies have shown that clinical spectrum of NMO includes cerebral and brainstem syndromes as well [1–3]. Brain lesions on magnetic resonance imaging (MRI) are found in the majority of NMO patients with long-standing disease [1, 2, 4–6], which makes it sometimes difficult to differentiate this disorder from multiple sclerosis (MS).

Ultrahigh-field MRI (4 Tesla or above) affords an unprecedented view of brain structures and pathology *in vivo* on a submillimeter scale due to high signal-to-noise ratio [7, 8]. The largely enhanced susceptibility of ultrahigh-field MR effect provides unique contrast between lesions and veins. This feature makes ultrahigh-field MR a valuable tool for differentiating plaques of MS, which frequently contain a venule, from lesions of other etiologies that rarely do [9].

Our aim in this work is to characterize brain lesions in patients with neuromyelitis optica spectrum disorders (NMOsd) using 7 T MR and to discuss the findings in the context of the growing literature on ultrahigh-field imaging in MS [9–19]. 

## 2. Methods

Inclusion criteria in the study were the diagnosis of NMO-spectrum disorders (NMOsd) defined either as NMO by 2006 Wingerchuk et al. criteria [20], or recurrent longitudinally extensive transverse myelitis (LETM) with NMO IgG seropositivity, or recurrent optic neuritis (ON) with NMO IgG seropositivity. NMO IgG was measured at the Mayo Clinic Laboratory (Rochester, MN) by either immunohistochemical or enzyme-linked immunosorbent assay [21]. All nonbedbound NMOsd patients followed by the neurologists of the NYU-MS Center (I. K. and J. H.) were invited to participate in the study. For each patient, we collected the following information: gender, ethnicity, country of birth, age at symptom onset, disease duration at MRI, index event, number of optic neuritis and of transverse myelitis relapses, visual acuity and ambulatory status at last followup, NMOsd therapy, and presence and number of vascular risk factors (defined as history of smoking, hypertension, hypercholesterolemia, or cardiovascular disease). 

Ultrahigh-field imaging was carried out on a 7-Tesla whole-body human MR system (MAGNETOM, Siemens Medical Solution, Erlangen, Germany) at the Center for Biomedical Imaging/Radiology at NYU Langone Medical Center, which uses gradients of 45 mT/m with maximum gradient strength of 72 mT/m effective and a slew rate of 200 T/m/s (346 T/m/s effective). The head coil was used with a birdcage-like circularly polarized transmit coil and a 24-element phased array coil located on a close-fitting helmet-like device (Nova Medical, Inc., MA). The imaging protocol consisted of high-resolution axial 2D high-resolution T2*-weighted gradient echo (GRE), T2-weighted turbo spin-echo (TSE), fluid-attenuated inversion recovery (FLAIR), and sagittal T1-weighted 3D magnetization-prepared rapid acquisition of gradient-echo (MPRAGE) imaging. No contrast agent was applied. The total scan time was 41 minutes and 23 seconds. The parameters of T2*-weighted gradient echo (GRE) include TR/TE/flip angle = 580 ms/25 ms/35° and slice thickness = 2 mm, with slice gap of 2.6 mm with pixel of 0.23 × 0.23 mm^2^, which were optimized for better signal-to-noise and contrast between lesions and venous structures. For axial T2-weighted TSE, the acquisition parameters were TR/TE/flip angle = 14300/73 ms/144°; slice thickness = 2 mm; FOV = 200 × 187 mm^2^; matrix = 384 × 384 mm^2^. For axial SPACE FLAIR imaging, the acquisition parameters were TR/TE/TI = 8000/380/2100 ms; voxel size = 1 × 1 × 1 mm^3^ isotropic. For sagittal T1-weighted 3D MPRAGE, the acquisition parameters were TR/TE/TI = 2250/2.37/1100 ms, section thickness = 1 mm; FOV = 256 × 256 mm^2^, matrix = 384 × 384.

Infratentorial volume could not be reliably evaluated because our 7 T receive coil yielded poor signal-to-noise ratio in this region, and the examination was therefore limited to supratentorial region. Two experienced observers (Y. G. and I. K.) examined T2-weighted and 2D gradient echo (T2*) imaging sequences for presence of lesions. To reduce the possibility of misidentification, only the lesions that were present on both sequences were counted. For each lesion, we recorded lesion size, configuration, location, and presence or absence of central venule and of hypointense peripheral ring on T2* sequence. Lesion localization was defined as periventricular (<2 mm of ventricular border), juxtacortical (<2 mm from cortical ribbon), subcortical/deep white matter (>2 mm from cortex or ventricle, excluding corpus callosum), corpus callosum, cortex, and deep grey matter nuclei. Lesions were described as “perivenous” if they were seen to be traversed by a vein on T2* sequence [9]. We also recorded the pattern of periependymal abnormalities.

Groups were compared using Mann-Witney *U* test for continuous variables. 

Data collection was carried out according to protocol approved by the Institutional Review Boards of NYU-Langone Medical Center, and all patients gave informed consent in writing before participation.

## 3. Results

Ten patients agreed to participate in the study, six of whom were diagnosed with NMO [20] and four with limited forms of NMO. All patients were women and NMO IgG-seropositive. Demographic, disease-related, and radiologic characteristics of our NMOsd cohort are shown in Table 1. 

**Figure 1:**
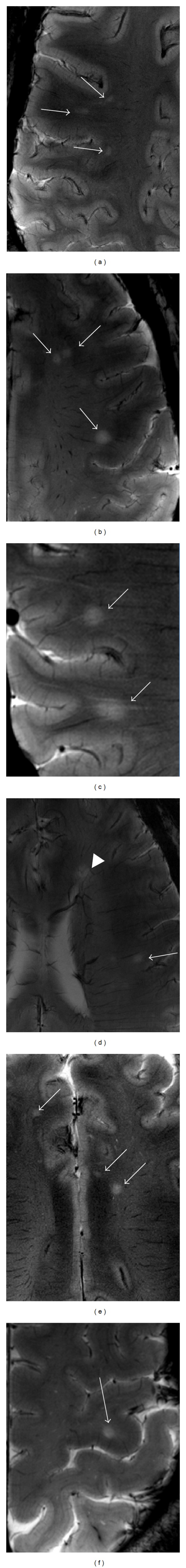
Examples of typical lesions in NMOsd. Brain lesions on T2*-weighted GRE images with in-plane resolution of 0.2 × 0.2 mm^2^ in six patients with NMO. Most lesions (approximately 85%) (arrows in (a), (b), (c), (d), and (e)) are small, round-shaped, and located in subcortical regions without typical sign of central venule or perivenous migration as seen in MS lesions. Only a few lesions have visible central veins (arrowhead in (d); this particular lesion was seen in enhance with gadolinium on a conventional brain MRI study performed three years previously). Only 3% lesions are juxtacortical (long arrow in (f)).

**Figure 2:**
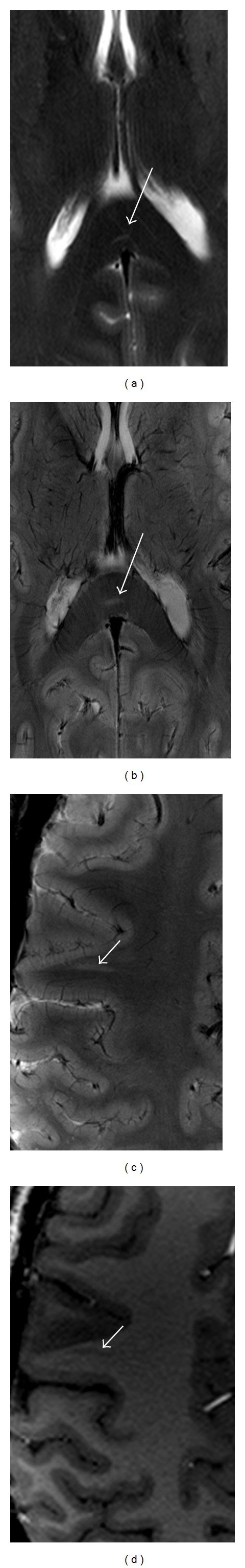
Examples of “thread-like” lesions in NMOsd. Thread-like lesions in two patients. A thread-like lesion along the corpus callosum white matter tract instead of venous course in one patient on T2-weighted (a) and T2*-weighted GRE (b) images (long arrows). Another lesion in subcortical region on T2*-weighted GRE (c) and T1-MPRAGE (d) images (short arrow) in the second patient.

**Figure 3:**
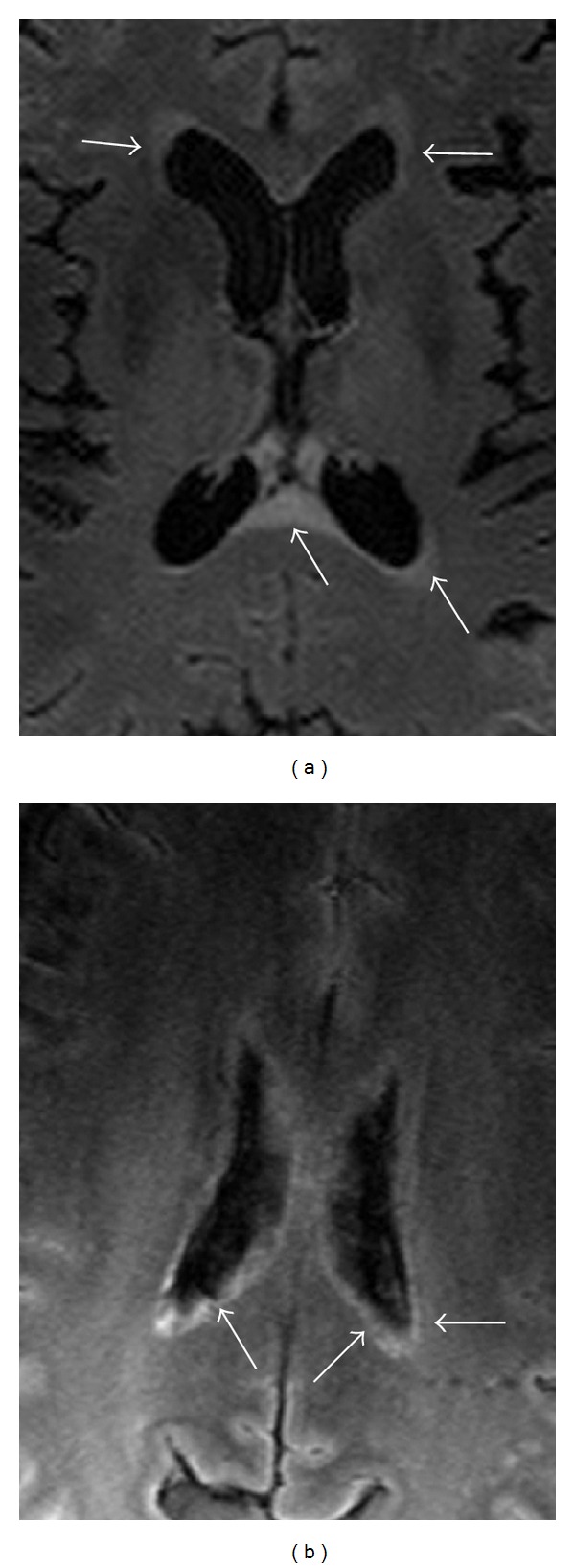
Examples of diffuse periependymal abnormalities. Abnormal and thickened signal intensities (arrows) along ependymal layers on FLAIR images in two patients ((a) and (b)).

We observed lesions in every patient. The total number of lesions was 92 (mean number was 9.2 ± 8.8; median: 6; range: 2–30). Seventy-six lesions (83%) were subcortical, 8 (9%) were juxtacortical, 3 lesions (3%) were found in corpus callosum, and 5 (5%) were periventricular (4 of which were adjacent to frontal horns of the lateral ventricles). We did not detect any lesions in the cortex or basal ganglia. All but three of the 92 lesions were <3 mm in cross-sectional diameter, and all lesions were <5 mm. The lesions generally appeared round on axial cross-sections (Figures 1(a)–1(f)). None evidenced hypointense ring on T2* sequence. We also observed several “thread-like” lesions, and these were invariably hypointense on T1-weighted sequences (Figures 2(a)–2(d)). Several lesions, which were <3 mm in cross-sectional diameter, could be seen on three consecutive slices, indicating that they were at least 6 mm long. In all, eight lesions (9%) were classified as “thread-like.” Two patients had diffuse periependymal abnormalities on T2-weighted sequences (Figures 3(a) and 3(b)). We did not observe any tumefactive or longitudinally extensive lesions in our patients.

Only eight lesions (9%) were traversed by a venule.  Figure 1(d) shows an example of such lesion. Three perivenous lesions were seen in one patient with NMO who had a total of four lesions, and five patients had one perivenous lesion each. 

The group with limited form of NMOsd, comprised of 4 patients, tended to have more lesions (mean: 13.4), compared to the six-patient NMO group (mean: 4.8), but the difference was not statistically significant (Mann-Whitney *U* test, *U* = 16.5  *P*-two tailed = 0.35). We did not observe a trend for increasing lesion number with age, disease duration, or the number of vascular risk factors (Table 1).

## 4. Discussion

T2-weighted hyperintense lesions on 7 T MRI were observed in all ten NMOsd patients in our cohort. All but three of the 92 lesions were <3 mm in largest diameter. Nearly all lesions were round (Figure 1), but several lesions had a distinct, “thread-like” appearance on axial cross-section (Figure 2). We consider it unlikely that “thread-like” lesions represent dilated Virchow-Robin spaces (VRS) because of their location, orientation, and signal characteristics. VRS are typically found in basal ganglia with penetrating arteries mostly from circle of Willis. They appear as round dots on axial plane with similar signal as CSF (i.e., brightly hyperintense spots on T2-weighted sequence) [22]. In contrast, “thread-like” lesions found in our NMO patients were observed mostly in juxtacortical white matter and corpus callosum, do not follow arterial course, and were not as bright as CSF on T2-weighted sequence. Thread-like lesion appearance has not, to our knowledge, been reported in other conditions, including MS, which is characterized by perivenous ovoid lesions [23].

With respect to lesion distribution, the overwhelming majority of lesions in NMOsd (~85%) were subcortical. The few discrete periventricular lesions were mostly located anterior to the frontal horns (Figure 1(d)) and were more extensive than the typical “periventricular caps” seen in healthy subjects [24]. Two of our patients exhibited diffuse periventricular abnormalities (Figure 3), which is notable in view of known rich expression of Aquaporin-4, the target of NMO IgG, in the ependymal layer [25]. In one patient (Figure 3(a)), splenium of the corpus callosum was affected in the “diffuse spreading” manner described as being characteristic of NMO in a Japanese cohort [26]. The discrepancy in the prevalences of diffuse spreading lesions in corpus callosum in our series (10%) and the Japanese series (71%) could perhaps be due to lesser degree of disability of our cohort, in which only 30% of patients required any kind of ambulatory assistance compared to the Japanese cohort, in which half of the patients needed ambulatory assistance. It is also possible that genetic background impacts phenotypic and radiographic expression of NMO. A patient from the Far East appears to manifest brain symptoms and large “NMO-specific” brain lesions more commonly than in the West [1, 2, 5].

Cortical lesions were not found in any of our patients in agreement with the recent radiographic studies that utilized double inversion recovery sequence [27] and ultrahigh-field MR [28] to image the cortex in NMO as well as a histopathologic study [29]. Absence of focal cortical lesions in NMOsd need not imply absence of other cortical pathology [30], but is helpful for differentiating NMO from MS, in which cortical lesions are increasingly recognized, especially with ultrahigh-field MR [11–14]. 

Importantly, only 9% of NMOsd brain lesions in our series contained a central vessel. This is in stark contrast with MS, in which 60–80% of cerebral lesions visualized with ultrahigh-field MRI are traversed by a venule [9, 10, 15, 16]. Paucity of intralesional venules in our series is not contradictory to results of a study in which venules were detected in a high proportion of white matter lesions of any etiology on susceptibility weighted imaging [31]. The latter study focused exclusively on lesions >3 mm in diameter, while nearly all lesions in our series were ≤3 mm in diameter. 

Absent histopathological correlation, it is impossible to be certain that brain lesions seen in our cohort are due to an NMO-specific pathogenic process. Some of the lesions could theoretically represent stigmata of “small vessel disease” (SVD), in itself a pathologically heterogeneous entity [32]. For a number of reasons, SVD origin of observed lesions seems to us to be less likely explanation. First, the finding of juxtacortical and corpus callosum lesions and the absence of basal ganglia lesions militate against SVD etiology. Secondly, “thread-like lesions” (Figures 2(a)–2(d)) have not been described in SVD. Third, we could find no correlation between vascular risk factors and the number of brain lesions (Table 1) and observed small lesions in patients in their 20s and 30s with no vascular risk factors. Lastly, in at least three of our patients, a lesion on 7 T MRI was seen to enhance with gadolinium previously on a conventional MRI study (unpublished data). This observation supports inflammatory etiology for at least some of the lesions herein described. Whatever the pathogenesis of small subcortical lesions NMO may be, these “non-specific” lesions represent the most consistent feature of NMO on brain MRI [5]. 

Our data is broadly consistent with recently published findings of 7 T MRI of the brain in 10 German NMOsd patients [28]. The German NMOsd cohort was somewhat older than ours (46 years versus 42 in our group) but had a slightly shorter disease duration (7 years versus 9 years). Both groups were exclusively female, and all but one patient were NMO IgG seropositive. In neither ours nor the German NMOsd cohort were any cortical lesions observed, and the lesions that were present were predominantly small and subcortical. The proportion of perivenous lesions among the German NMOsd patients was higher than in our group (35% versus 9%), which may be related to the higher average number of lesions in their cohort (14 lesions versus 9 lesions in our group). Higher lesion number increases the probability of chance finding of a traversing vein. Importantly, in both cohorts, the number of perivenous lesions was significantly smaller than what is typically reported in MS (>80%) [8]. Finally, although hypointense ring, which is indicative of iron or hemosiderin deposition, is found in a significant minority of MS lesions [17, 28], it was only observed in one out of ten NMOsd patients in the German NMO cohort and none of our ten patients. This further supports differential brain lesion pathogenesis in MS and NMO. 

Our work and that of Sinnecker et al. [28] demonstrate that small round subcortical lesion without a central venule or hypointense ring is the most characteristic feature on NMOsd on ultrahigh-field MRI. In contrast, the signature lesion of MS is oblong, periventricular, and traversed centrally by a venule [9, 10, 15, 16], corresponding to “Dawson's fingers”, described pathologically almost a hundred years ago [33]. We suggest that the absence of “Dawson's fingers” and cortical lesions on 7 T MRI in a patient with multiple, >9, T2 hyperintense lesions should alert the radiologist that diagnosis of MS is unlikely. 

Ultrahigh-field imaging of brain in NMOsd provides strong supportive evidence that MS and NMO constitute two distinct nosological entities despite their predilection for the same organs. Ultrahigh-field brain MRI may prove useful for differentiating NMOsd from MS.

## Figures and Tables

**Table 1:** Demographic, disease-related, and radiographic characteristics of the NMOsd cohort.

Patient number	1	2	3	4	5	6	7	8	9	10		Cohort
Clinical												
Ethnicity/race	Hispanic	Multiethnic^1^	African-Amer.	Hispanic	Hispanic	Caucasian	Hispanic	Afro-Carribian	Caucasian	Hispanic	Non-Caucasian %	80%
Country of birth	Domin. Rep.	USA	USA	Equador	Columbia	USA	Peru	West Indies	Latvia	Domin. Rep	Non-US born %	70%
Age at onset	28	19	27	38	46	46	38	60	56	63	Mean age onset (st. dev), yrs	42.1 (±14.8)
Age at time of MRI (yrs)	30	33	37	41	53	54	60	61	69	75	Mean age at MRI (st. dev), yrs	51.3 (±15.4)
Index event	TM	TM	ON	ON	ON	TM	TM	ON	TM	ON	Onset event ON %	50%
Optic Neuritis	2	5	6	2	3	0	3	2	0	1	ON, median (range)	2 (0–6)
Myelitis	3	4	4	0	1	2	5	0	4	1	Myelitis, median (range)	2 (0–5)
Form of NMO	NMO	NMO	NMO	Recurrent ON	NMO	Recurrent TM	NMO	Recurrent ON	Recurrent TM	NMO	NMO (Mayo 2006 criteria) %	60%
Visual acuity	<20/100 OS	<20/100 OU	<20/100 OU	20/80 OD	<20/100 OS	≤20/30 OU	<20/100 OS	<20/100 OS	≤20/30 OU	≤20/30 OU	Visual impaired % ≥1 eye	70%
Need ambulatory assistance	Cane	Walker	Unassisted	Unassisted	Unassisted	Unassisted	Wheelchair	Unassisted	Unassisted	Unassisted	Need ambulatory assist %	30%
Current NMO treatment	Mycophenolate	Mycophenolate prednisone	Rituximab, methotrexate	Azathioprine	Mycophenolate prednisone	Azathioprine	Rituximab	Azathioprine	Prior cytoxan	Azathioprine	On NMO therapy %	90%
Vascular risk factors	No	No	No	No	No	HTN, HLD	HLD	HTN	No	HTN, HLD	With vascular risk factors %	40%
MRI (lesion number on T2*/T2)												
Subcortical	7	4	4	2	4	15	7	7	24	2	Subcortical, mean (st dev)	7.6 (±6.9)
Periventricular	0	1	1	0	0	1	2	0	0	0	Periventricular, mean (st dev)	0.5 (±0.7)
Juxtacortical	0	0	0	0	0	2	0	0	6	0	Juxtacortical, mean (st dev)	0.8 (±1.9)
Corpus callosum	1	0	0	0	0	1	1	0	0	0	Corpus callos., mean (st dev)	0.3 (±0.5)
“Thread-like”	3	0	3	0	0	1	0	1	0	0	“Thread-like,” mean (st dev)	0.8 (±1.2)
Perivenous	0	1	1	0	3	0	1	1	0	1	Perivenous, mean (st dev)	0.8 (±0.9)

Total, #	8	5	5	2	4	19	10	7	30	2	Total lesion count, mean (st dev)	9.2 (±8.8)

^1^Native American/Irish/African-American.

All lesions were <3 mm in diameter, except for 3 lesions in patient number 9 (see Table 1), which were ~5 mm in diameter. No cortical or basal ganglia lesions were seen.

HTN: hypertension; HLD: hyperlipidemia; NMO: neuromyelitis, optica; OD: right eye; OS: left eye; OU: both eyes; ON: optic neuritis; TM: transverse myelitis.
